# NR1D1 modulates synovial inflammation and bone destruction in rheumatoid arthritis

**DOI:** 10.1038/s41419-020-2314-6

**Published:** 2020-02-18

**Authors:** Hui Liu, Yuanli Zhu, Yutong Gao, Dahu Qi, Liming Zhao, Libo Zhao, Changyu Liu, Tenghui Tao, Chuankun Zhou, Xuying Sun, Fengjing Guo, Jun Xiao

**Affiliations:** 1 Department of Orthopedics, Tongji Hospital, Tongji Medical College, Huazhong University of Science and Technology, Wuhan, 430030 China; 2 Institute of Pathology, Tongji Hospital, Tongji Medical College, Huazhong University of Science and Technology, Wuhan, 430030 China; 3 Department of Pathology, Union Hospital, Tongji Medical College, Huazhong University of Science and Technology, Wuhan, China

**Keywords:** Rheumatoid arthritis, Chronic inflammation

## Abstract

Rheumatoid arthritis (RA) is a chronic autoimmune disease characterized by synovial hyperplasia, pannus formation, and cartilage and bone destruction. Nuclear receptor subfamily 1 group D member 1 (NR1D1) functions as a transcriptional repressor and plays a vital role in inflammatory reactions. However, whether NR1D1 is involved in synovial inflammation and joint destruction during the pathogenesis of RA is unknown. In this study, we found that NR1D1 expression was increased in synovial tissues from patients with RA and decreased in RA Fibroblast-like synoviocytes (FLSs) stimulated with IL-1β in vitro. We showed that NR1D1 activation decreased the expression of proinflammatory cytokines and matrix metalloproteinases (MMPs), while NR1D1 silencing exerted the opposite effect. Furthermore, NR1D1 activation reduced reactive oxygen species (ROS) generation and increased the production of nuclear transcription factor E2-related factor 2 (Nrf2)-associated enzymes. Mitogen-activated protein kinase (MAPK) and nuclear factor κB (NF-κB) pathways were blocked by the NR1D1 agonist SR9009 but activated by NR1D1 silencing. NR1D1 activation also inhibited M1 macrophage polarization and suppressed osteoclastogenesis and osteoclast-related genes expression. Treatment with NR1D1 agonist SR9009 in collagen-induced arthritis (CIA) mouse significantly suppressed the hyperplasia of synovial, infiltration of inflammatory cell and destruction of cartilage and bone. Our findings demonstrate an important role for NR1D1 in RA and suggest its therapeutic potential.

## Introduction

Rheumatoid arthritis (RA) is a chronic complicated autoimmune disease characterized by synovial hyperplasia, pannus formation, and bone and cartilage destruction. Patients with RA typically experience a chronic fluctuating disease course, eventually resulting in loss of function and malformation of joints, which markedly reduces the quality of life^1,2^. However, the cells and molecular pathogenesis of RA is complex and unclear. Fibroblast-like synoviocytes (FLSs), T lymphocytes, B lymphocytes, monocytes, and neutrophils drive the progression of RA, particularly FLSs and macrophages^3,4^. Accumulating research indicate that FLSs play vital role in RA by producing proinflammatory cytokines, such as interleukin (IL)-6, IL-1β, tumor necrosis factor (TNF)-α, inducible nitric oxide synthase (iNOS), and proteinases, such as matrix metalloproteinases (MMPs), which perpetuate joint destruction^3,5^. In addition, macrophages can differentiate into osteoclasts, thus regulating inflammation and bone turnover. Abnormal differentiation and activation of macrophages promotes RA progression by contributing to chronic inflammation and bone destruction. Activated macrophages drive the progression of RA by producing proinflammatory cytokines and chemokines, evoking the destruction of articular cartilage and subchondral bone^6,7^. Therapies for RA inhibit the inflammatory cascade (non-steroidal anti-inflammatory drugs, NSAIDs) or slow disease progression (disease-modifying antirheumatic drugs, DMARDs)^8^. Although those agents can alleviate symptoms, their side effects are considerable. Therefore, identification of a factor with anti-inflammatory and anti-bone destruction activity would promote the development of novel agents for RA.

NR1D1, also known as REV-ERBα, is a member of the nuclear receptor 1D subfamily and functions as a transcriptional repressor^9,10^. It inhibits transcription of a target gene by binding to the REV-ERB response element. NR1D1 is implicated in the circadian rhythm because its deletion results in interruption of the circadian rhythm in mice. In addition, NR1D1 suppresses the feedback loop of BMAL1 to consolidate the rate of the day/night oscillator^11^. NR1D1 also regulates the expression of metabolism-related genes, thereby linking the circadian rhythm to cellular metabolism^12^. Studies have shown that NR1D1 is closely related to various physiological processes of cells, including cell differentiation, metabolism, mitochondrial biosynthesis, and inflammatory responses, making it a potential therapeutic target for cancer, dyslipidemia, and inflammatory diseases^13–15^.

Recent research has shown that NR1D1 modulates experimental colitis via the NF-κB/NLR family pyrin domain-containing 3 (NLRP3) axis^16^. Griffin and colleagues found that activation of NR1D1 inhibited lipopolysaccharide (LPS)-induced microglial activation and suppressed the development of neurogenic inflammation^17^. In addition, NR1D1 regulates the development of Th17 cells and Th17 cell-mediated autoimmune diseases^18^. NR1D1 is linked to the immune system, as it has been shown to repress Toll-like receptor 4, Cx3cr1, and IL-6 expression in macrophages^15^. Therefore, activated NR1D1 alleviates the progression of pneumonia and fulminant hepatitis^19,20^. NR1D1 regulates the inflammatory response; however, whether NR1D1 mediates the pathogenesis of RA and related mechanisms remain poorly defined.

In this study, we conducted a systematic study of the role of NR1D1 in synovial inflammation and joint destruction and the related mechanisms during the pathogenesis of RA.

## Results

### NR1D1 expression in RA synovial tissues and cells

**Fig. 1:**
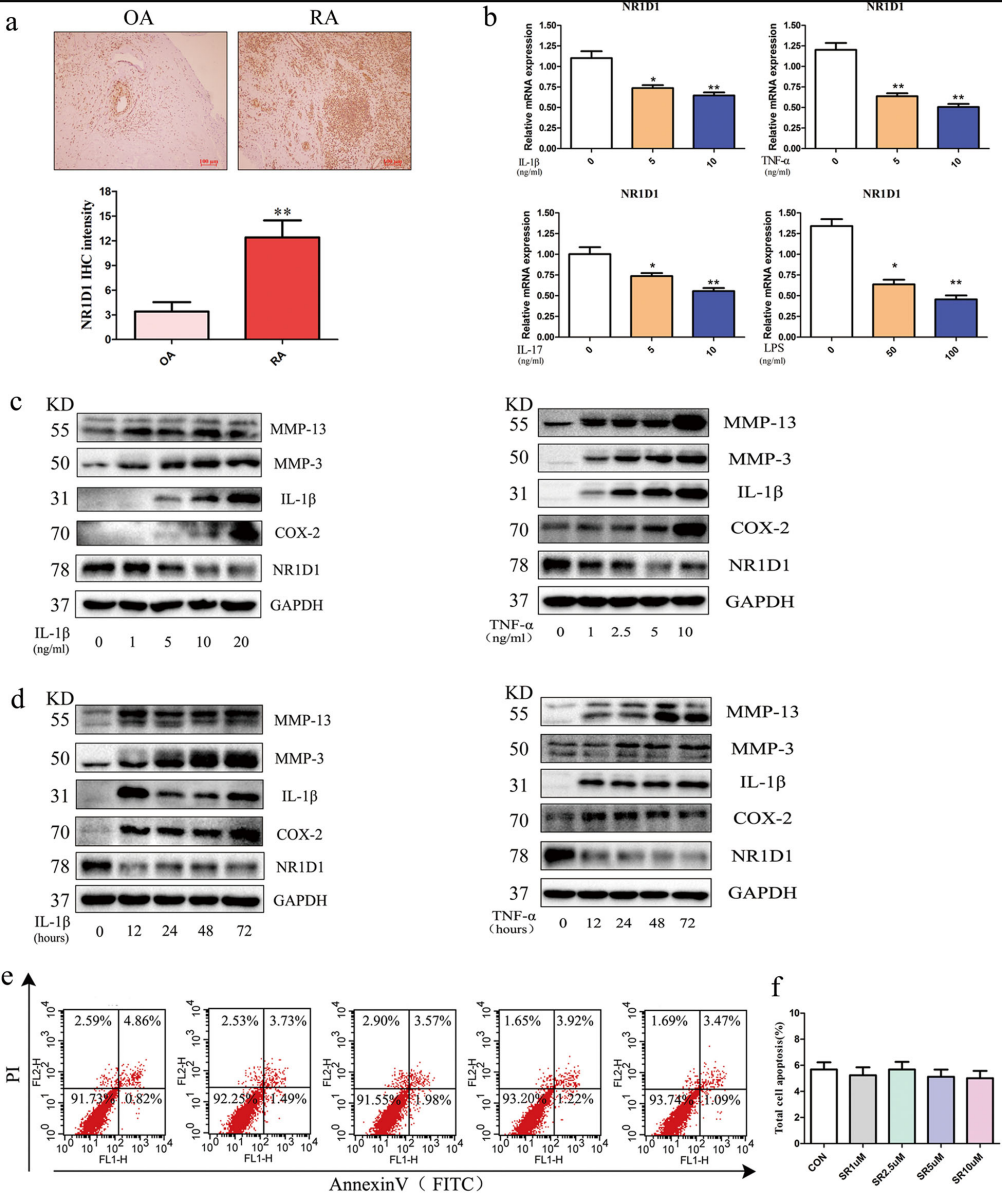
Expression of NR1D1 in synovial tissue and FLSs of patients with RA. **a** NR1D1 expression in synovial tissues. Representative images of immunohistochemical staining of tissues from patients with RA (*n* = 8) and OA (*n* = 4). Quantitative analyses (below) and representative images (upper, original magnification 10×, scale bar = 100 μm). **b** RA FLSs were incubated with IL-1β, TNF-α, IL-17, and LPS for 24 h (left). The NR1D1 mRNA level was determined by qRT-PCR. Data are means ± SEM of three independent experiments. *p < 0.05, **p < 0.01 versus the control group. **c** Representative western blots of NR1D1, IL-1β, COX-2, MMP3, and MMP13 in RA FLSs stimulated with IL-1β or TNF-α at the indicated concentrations for 72 h. **d** Representative western blots of NR1D1, IL-1β, COX-2, MMP3, and MMP13 in RA FLSs stimulated with IL-1β or TNF-α for the indicated times. **e**, **f** Flow cytometric analysis of apoptosis. RA FLSs were exposed to SR9009 (1–10 μM) for 24 h, stained using an Annexin V/PI apoptosis kit, and subjected to flow cytometry. Data are means ± SEM of three independent experiments; *p < 0.05 versus the control group.

To assess the role of NR1D1 in the progression of RA, we detected NR1D1 in RA and OA synovial tissues by immunohistochemical staining. NR1D1 staining was evident in RA synovial tissues; by contrast, minimal staining was observed in OA synovial tissues (Fig. 1a). RT-qPCR showed that the mRNA level of NR1D1 in FLSs was decreased by IL-1β, TNF-α, IL-17, and LPS. (Fig. 1b). Western blotting showed that the protein levels of COX-2, IL-1β, MMP3, and MMP13 were elevated, while that of NR1D1 was consistent with its mRNA level (Fig. 1c, d). Annexin V/PI double staining was performed to assess the proapoptotic effect of the NR1D1 agonist SR9009 on RA FLSs; SR9009 had no obvious pro-apoptotic effect on cells (Fig. 1e, f).

### NR1D1 regulates the inflammatory response in RA FLSs

Proinflammatory cytokine production by FLSs plays a crucial role in the pathogenesis and progression of RA. Suppression of inflammatory cytokine production by monoclonal antibodies against IL-1β and TNF-α is effective against RA^3^. We first evaluated the toxicity of the NR1D1 agonist SR9009 in RA FLSs by CCK-8 assay. SR9009 at the highest dose tested (10 μM) did not exert detectable cytotoxicity (Fig. S1).

**Fig. 2:**
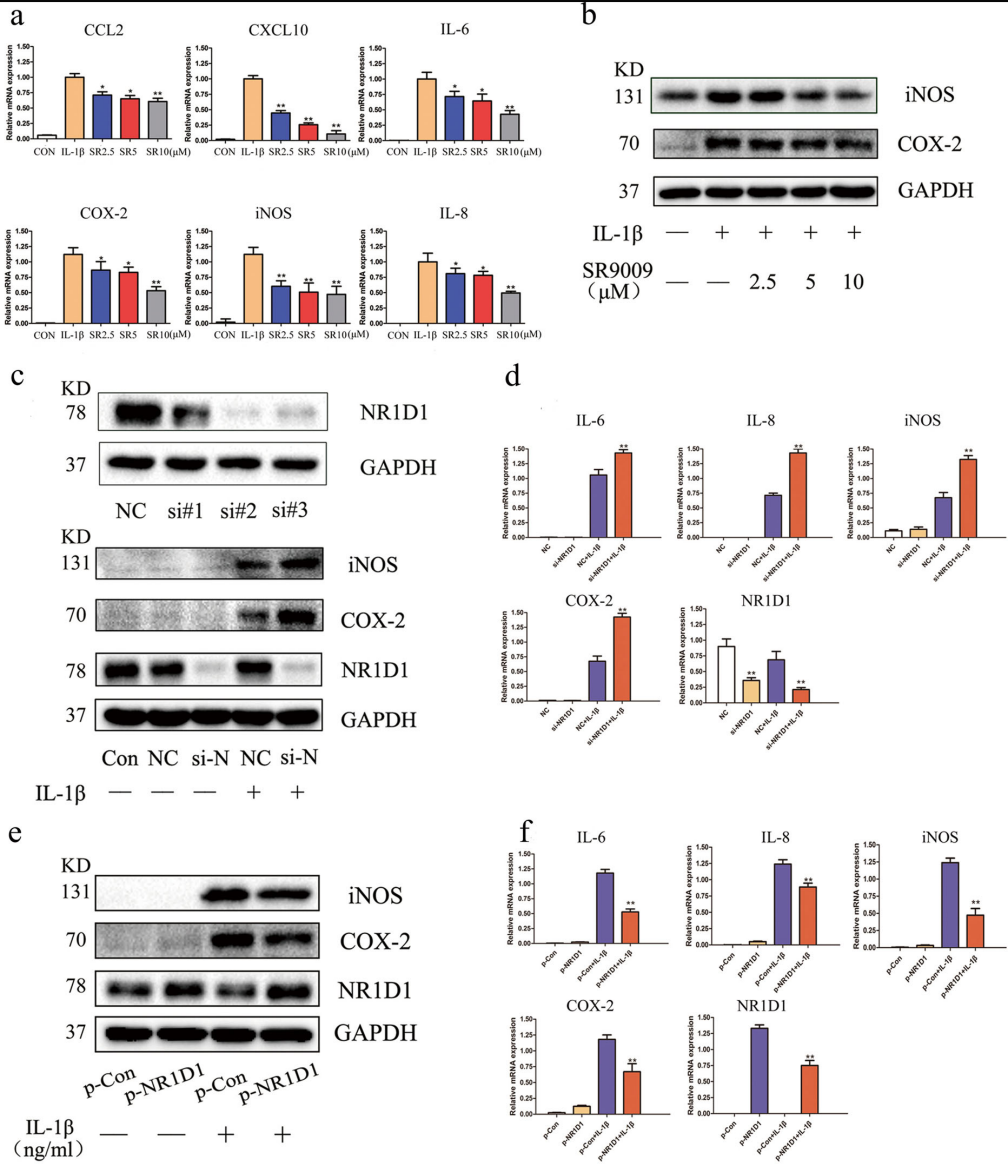
NR1D1 regulates the inflammatory response in RA FLSs. **a** IL-1β-mediated production of CCL2, CXCL10, IL-6, COX-2, iNOS, and IL-8 was diminished by SR9009, as determined by RT-PCR. Data are means ± SEM of three independent experiments. *p < 0.05, **p < 0.01 versus cells stimulated with only IL-1β. **b** COX-2 and iNOS levels were elevated by IL-1β but suppressed by SR9009, as determined by western blotting. **c** Efficacy of siRNA transfection. Total protein was extracted 72 h after transfection of RA FLSs with siRNA, and western blotting was performed (top). RA FLSs transfected with NR1D1 siRNA#2 exhibited increased COX-2 and iNOS levels after incubation with IL-1β (10 ng/mL). RA FLSs were transfected with NR1D1 siRNA and stimulated with IL-1β (10 ng/mL) for 72 h (bottom). **d** Expression of IL-6, IL-8, COX-2, and iNOS was increased in RA FLSs transfected with NR1D1 siRNA after incubation with IL-1β (10 ng/mL), as determined by RT-PCR. Data are means ± SEM of three independent experiments. *p < 0.05, **p < 0.01 versus negative control (NC) IL-1β-stimulated cells. **e** RA FLSs transfected with an NR1D1 overexpression plasmid exhibited decreased COX-2 and iNOS levels after incubation with IL-1β (10 ng/mL) as determined by western blotting. **f** Expression of IL-6, IL-8, COX-2, and iNOS decreased in RA FLSs transfected with an NR1D1 overexpression plasmid after incubation with IL-1β (10 ng/mL) as determined by RT-PCR. Data are means ± SEM of three independent experiments. *p < 0.05, **p < 0.01 versus empty plasmid and IL-1β-stimulated cells. si-N, si-NR1D1.

To evaluate the effect of SR9009 on proinflammatory cytokines, we measured the mRNA levels of IL-6, IL-8, COX-2, iNOS, chemokine (C-C motif) ligand 2 (CCL2), and C-X-C motif chemokine ligand 10 (CXCL10) in IL-1β-stimulated RA FLSs by real-time PCR. As shown in Fig. 2a, the mRNA levels of these proinflammatory cytokines were significantly upregulated by IL-1β but decreased by SR9009 in a concentration-dependent manner. Western blotting showed that SR9009 reduced the protein levels of COX-2 and iNOS (Fig. 2b). We next examined the severity of inflammation in vitro by altering NR1D1 expression. We used an siRNA to silence NR1D1 expression in RA FLSs. NR1D1-silenced RA FLSs showed increased protein levels of COX-2 and iNOS and increased mRNA levels of the proinflammatory cytokines IL-6, IL-8, COX-2, and iNOS (Fig. 2c, d). Furthermore, we assessed the effect of NR1D1 overexpression in RA FLSs by transfecting a plasmid carrying human NR1D1. NR1D1 overexpression significantly decreased the protein levels of IL-6, IL-8, COX-2, and iNOS (Fig. 2e, f).

### NR1D1 regulates MMP3 and MMP13 activation in RA FLSs

**Fig. 3:**
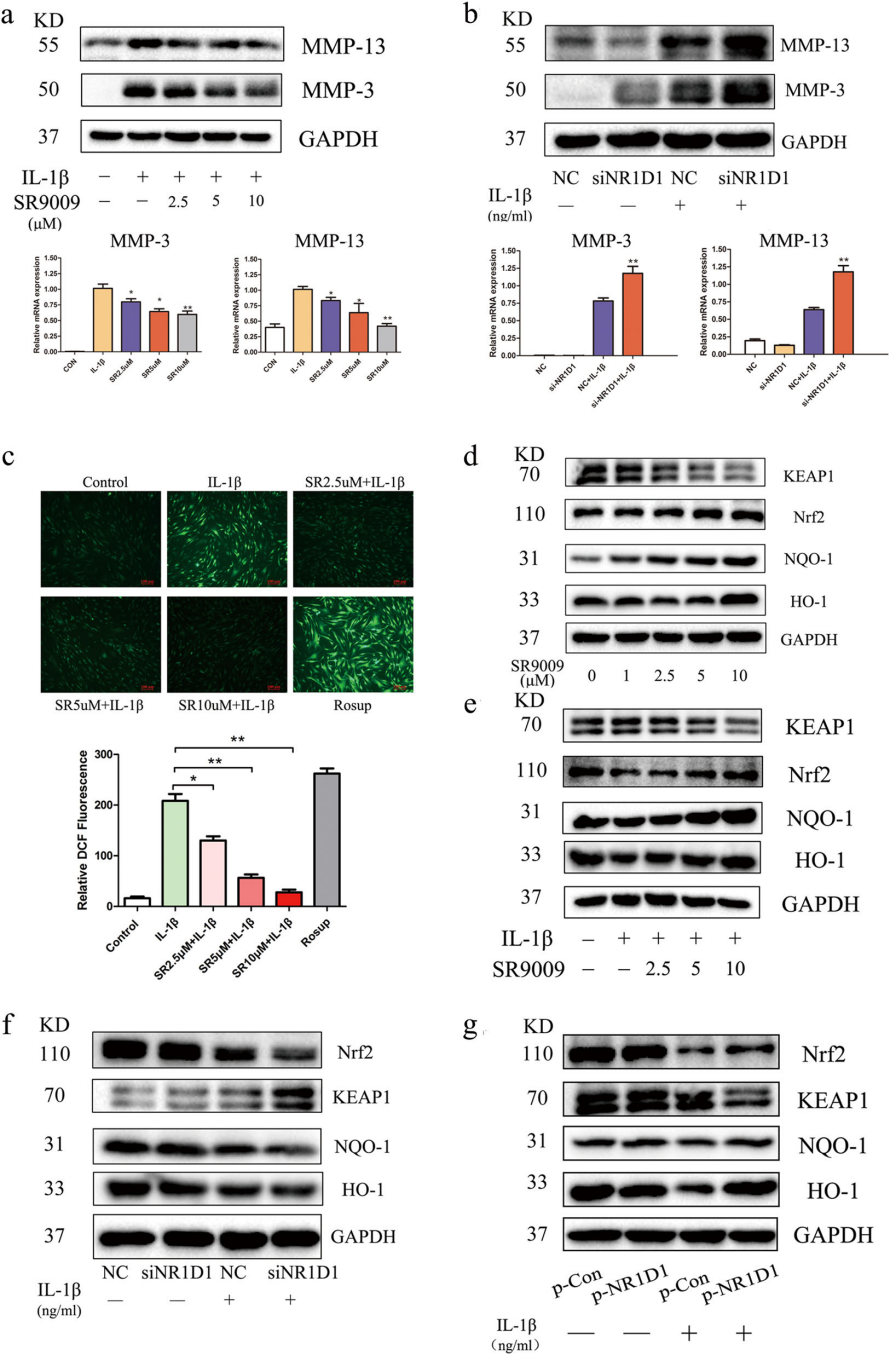
NR1D1 regulates MMP activation and the levels of Nrf2-associated enzymes in RA FLSs. **a** MMP13 and MMP3 levels were elevated by IL-1β but suppressed by SR9009, as determined by western blotting (top). IL-1β-mediated production of MMP13 and MMP3 was diminished by SR9009, as determined by RT-PCR (bottom). Data are means ± SEM of three independent experiments. *p < 0.05, **p < 0.01 versus cells stimulated with only IL-1β. **b** RA FLSs transfected with NR1D1 siRNA exhibited increased MMP13 and MMP3 levels after incubation with IL-1β (10 ng/mL) as determined by western blotting (top). Expression of MMP13 and MMP3 was increased in RA FLSs transfected with an NR1D1 siRNA after incubation with IL-1β (10 ng/mL) as determined by RT-PCR (bottom). Data are means ± SEM of three independent experiments. *p < 0.05, **p < 0.01 versus NC group IL-1β-stimulated cells. **c** RA FLSs were treated with SR9009 at the indicated concentrations for 1 h, stimulated with IL-1β (10 ng/mL) for 24 h, and the intracellular ROS level was analyzed by fluorescence microscopy. Representative images (top, original magnification 10×, scale bar = 100 μm) and quantitative analysis of the intracellular ROS level are shown (bottom). Data are means ± SEM of three independent experiments. *p < 0.05, **p < 0.01 versus cells stimulated with only IL-1β. **d**, **e** RA FLSs were treated with the indicated concentrations of SR9009 for 24 h with or without IL-1β, total lysates were prepared, and the levels of HO-1, NQO1, KEAP1, and Nrf2 were determined by western blotting. **f** Expression of HO-1, NQO1, KEAP1, and Nrf2 was decreased in RA FLSs transfected with NR1D1 siRNA and incubated with IL-1β (10 ng/mL) as determined by western blotting (left). **g** RA FLSs transfected with an NR1D1 overexpression plasmid exhibited increased levels of HO-1, NQO1, KEAP1, and Nrf2 after incubation with IL-1β (10 ng/mL) as determined by western blotting (right).

Cartilage destruction is a characteristic of RA, in which MMPs secreted by RA FLSs play an important role^21^. Therefore, we researched the effect of NR1D1 on the activation of MMP3 and MMP13. As shown in Fig. 3a, the protein and mRNA levels of MMP3 and MMP13 were markedly reduced by SR9009 in a concentration-dependent manner. In addition, NR1D1 silencing increased the protein and mRNA levels of MMP3 and MMP13 (Fig. 3b). These results demonstrated that NR1D1 regulates MMP3 and MMP13 expression and that activation of NR1D1 improves the destruction of RA cartilage by inhibiting MMP3 and MMP13 expression.

### NR1D1 regulates ROS generation and the production of Nrf2-associated enzymes in RA FLSs

Oxidative stress stimulates the production of several proinflammatory cytokines and inflammatory mediators, which causes oxidative stress and breakdown of cartilage and bone, exacerbating RA^22^. Upregulation of the intracellular ROS level plays a crucial role in the above, so we investigated the level of ROS in RA FLSs treated with SR9009 by quantifying DCFDA-positive cells. The results showed that SR9009 significantly reduced ROS production in RA FLSs (Fig. 3c). Nrf2 is a nuclear transcription factor that plays an important role in cytoprotective responses to oxidative stress. Therefore, we determined the effect of SR9009 on the levels of Nrf2-associated enzymes. As shown in Fig. 3d, e, SR9009 increased the level of HO-1, NQO-1, and Nrf2, which are Nrf2-dependent cytoprotective enzymes, and decreased that of KEAP1, which sequesters Nrf2 in the cytosol, irrespective of IL-1β stimulation. In addition, considering their key role in RA progression, the effect of NR1D1 on the levels of Nrf2-associated enzymes was evaluated. NR1D1-silenced RA FLSs stimulated with IL-1β exhibited reduced protein levels of HO-1, NQO-1, and Nrf2 and an increased protein level of KEAP1 (Fig. 3f). NR1D1 overexpression obviously increased HO-1, NQO-1, and Nrf2 protein levels, and markedly decreased that of KEAP1 (Fig. 3g). Therefore, activated NR1D1 reduced ROS generation by regulating the synthesis of Nrf2- associated enzymes.

### The NR1D1 agonist SR9009 inhibits the MAPK and NF-κB pathways

**Fig. 4:**
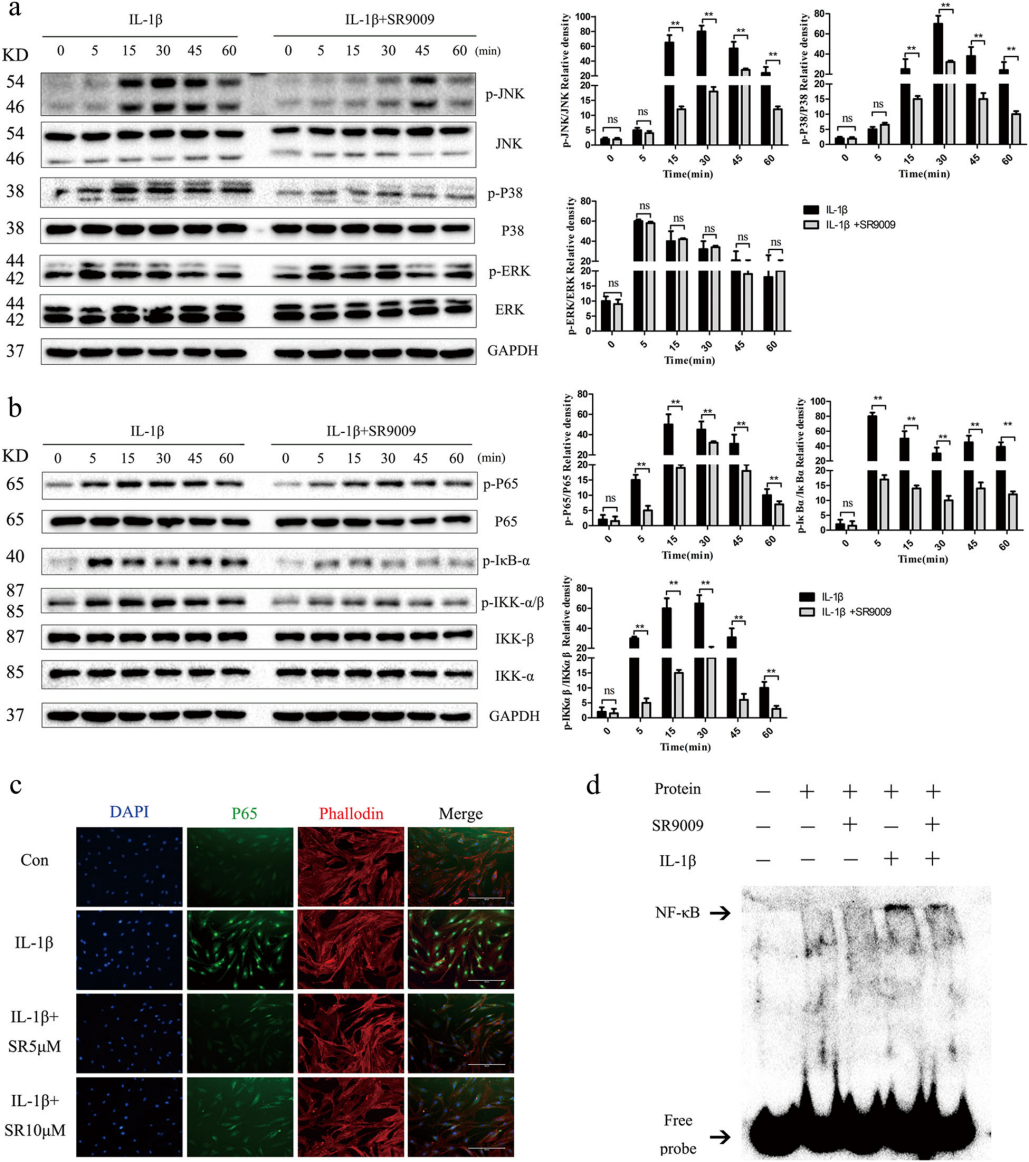
SR9009 suppresses activation of the MAPK and NF-κB signaling pathways. **a**, **b** RA FLSs pretreated with SR9009 (10 μM) for 12 h were stimulated with IL-1β for the indicated times. Representative images of the effect of SR9009 on the phosphorylation of p38, JNK, ERK, p65, IKKα/β, and IκBα (left). Right panel, densitometric analysis of a representative immunoblot from three independent experiments. *p < 0.05, **p < 0.01 versus cells stimulated with only IL-1β. **c** Effect of SR9009 on the nuclear translocation of NF-κB p65. Representative fluorescence micrographs of the effect of SR9009 on IL-1β-induced translocation of p65 (green) from three independent experiments (original magnification 10×, scale bar = 200 μm). **d** Binding of NF-κB to DNA as determined by EMSA. RA FLSs were pretreated with vehicle or 10 μM SR9009 for 24 h and stimulated with 10 ng/mL IL-1β or vehicle. ns, no significance.

**Fig. 5:**
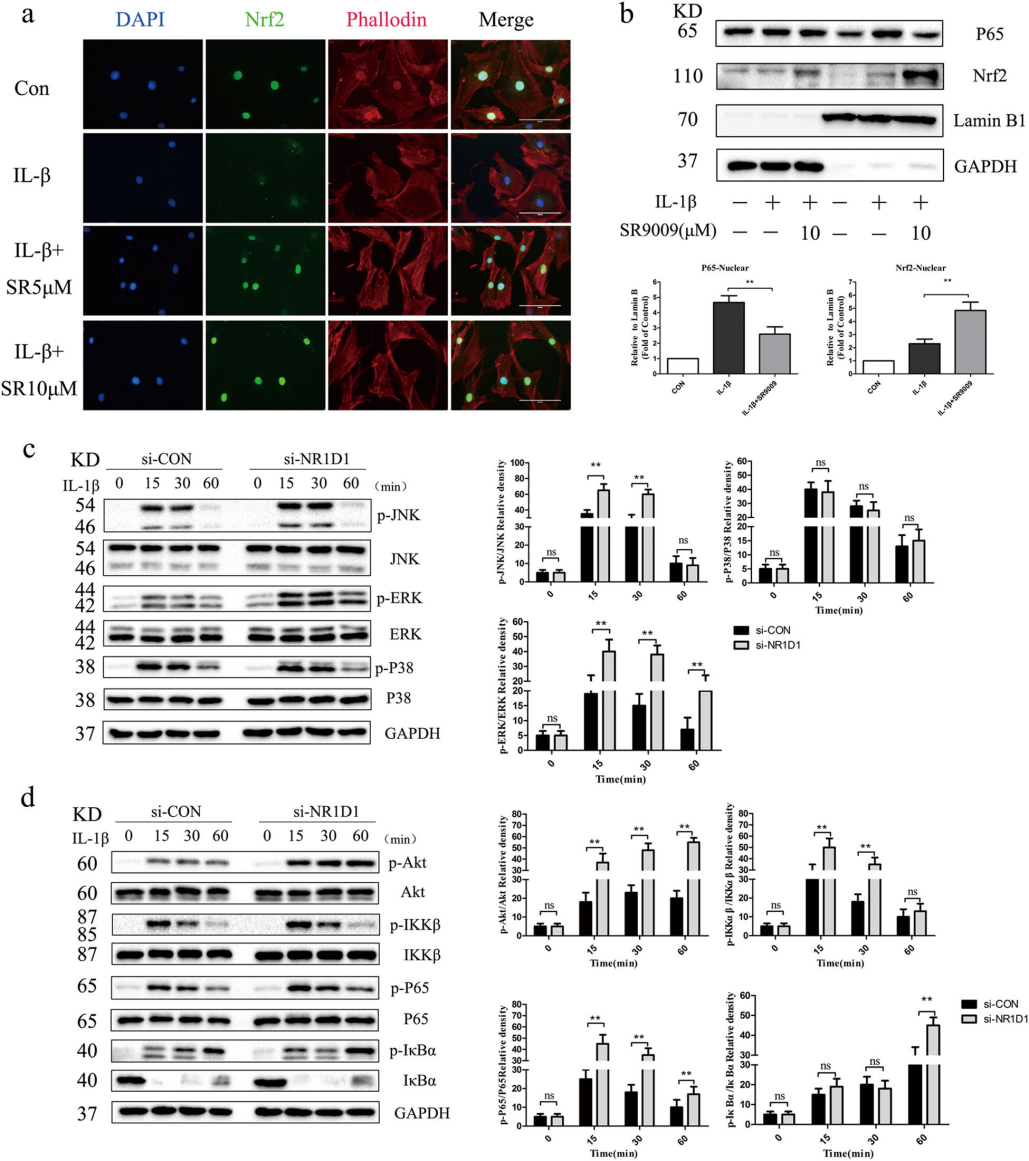
NR1D1 silencing activated MAPK and NF-κB signaling pathways. **a** Effect of SR9009 on the nuclear translocation of Nrf2. Representative fluorescence micrographs of the effect of SR9009 on IL-1β-induced translocation of Nrf2 (green) from three independent experiments (original magnification 20×, scale bar = 100 μm). **b** RA FLSs were treated with SR9009 at the indicated concentrations for 12 h and stimulated with IL-1β (10 ng/mL) for 30 min. Nuclear extracts of RA FLSs were prepared. Protein levels of p65, Nrf2, GAPDH, and lamin B as determined by western blotting (top) and densitometric analysis of an immunoblot from three independent experiments (bottom). *p < 0.05, **p < 0.01 versus cells stimulated with only IL-1β. **c**, **d** RA FLSs were transfected with an NR1D1 siRNA and stimulated with IL-1β for the indicated times. Representative images of the effect of NR1D1 silencing on the phosphorylation of p38, JNK, ERK, p65, IKKα/β, Akt, and IκBα (left). Right panel, densitometric analysis of an immunoblot from three independent experiments (right). *p < 0.05, **p < 0.01 versus cells stimulated with only IL-1β. ns, no significance.

The MAPK and NF-κB signaling pathways are involved in modulating synovial inflammation and joint destruction in RA^23,24^. To determine whether NR1D1 influences activation of the MAPK and NF-κB pathways, we first evaluated the effect of SR9009. As shown in Fig. 4a, the phosphorylated JNK level in IL-1β-stimulated RA FLSs was decreased by SR9009. In addition, SR9009 inhibited the IL-1β-induced phosphorylation of P38 but did not impact that of ERK. Next, we evaluated the role of SR9009 in regulating activation of the NF-κB pathway. Phosphorylation of p65, IKKβ, and IκBα was robustly induced by IL-1β, but was suppressed by SR9009 (Fig. 4b). Furthermore, SR9009 reduced p65 nuclear translocation and increased that of Nrf2 in RA FLSs, compared with IL-1β alone (Figs. 4c, 5a, b). The EMSA results also indicated a reduction in combination with transcription factor NF-κB in RA FLSs treated with SR9009 (Fig. 4d).

### NR1D1 silencing activates the MAPK and NF-κB signaling pathways

We further investigated whether NR1D1 silencing is involved in activation of the NF-κB and MAPK pathways in RA FLSs. As shown in Fig. 5c, NR1D1 silencing promoted IL-1β-induced phosphorylation of JNK and ERK but did not impact that of P38. Also, NR1D1 silencing increased IL-1β-induced phosphorylation of Akt, p65, IKKβ, and IκBα compared with IL-1β alone (Fig. 5d).

### SR9009 inhibits M1 macrophage polarization and suppresses osteoclastogenesis

**Fig. 6:**
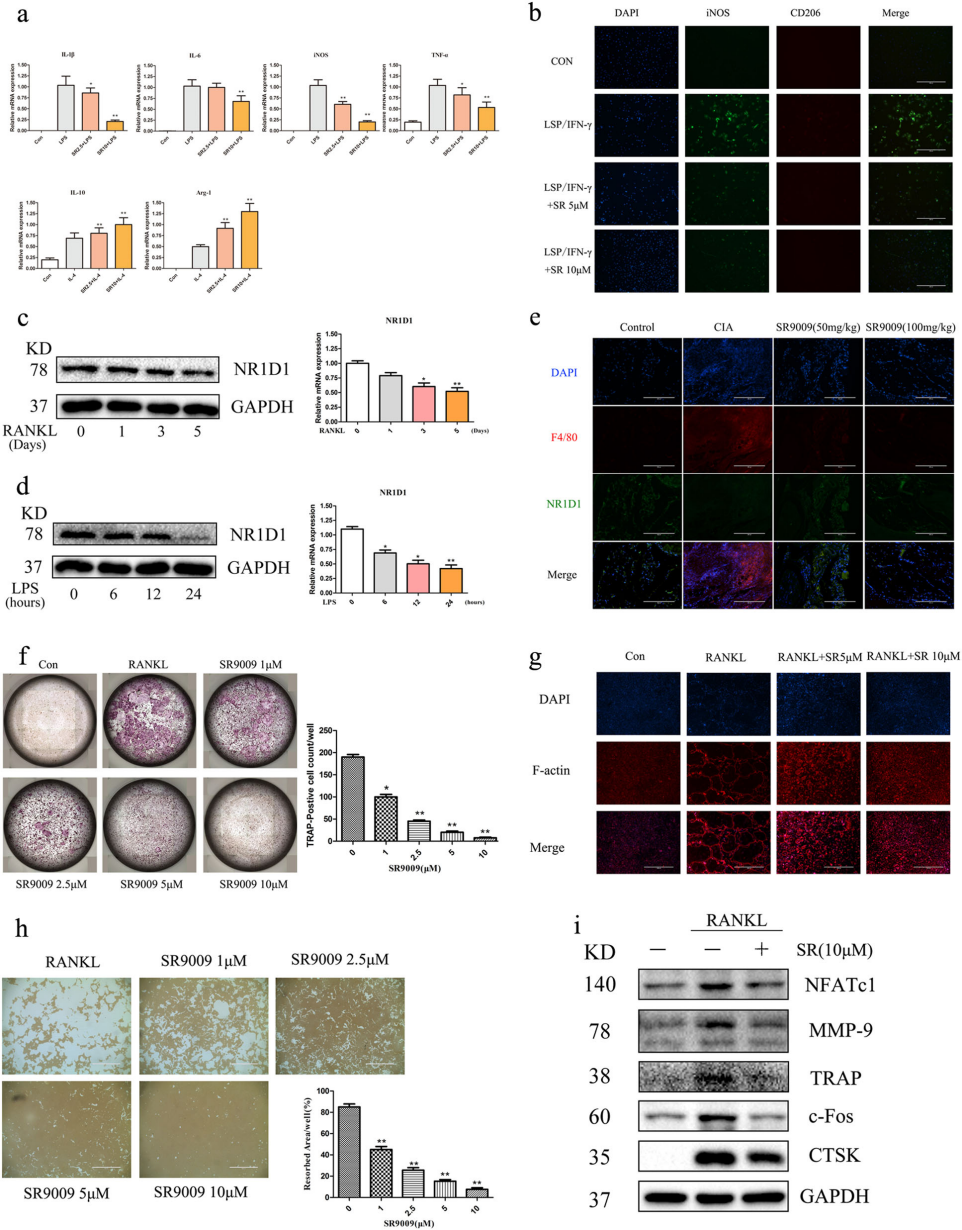
SR9009 inhibits M1 macrophage polarization and suppresses osteoclastogenesis. **a** Real-time PCR showing induction of M1 or M2 markers in BMMs stimulated with LPS (100 ng/mL) + IFN-γ (20 ng/mL) or with IL-4 (20 ng/mL) with or without SR9009. *p < 0.05, **p < 0.01 versus cells stimulated with only LPS or IL-4. **b** BMMs were subjected to immunofluorescence staining with anti-iNOS and anti-CD206 antibodies (original magnification 10×, scale bar = 400 μm). **c** The expression of NR1D1 during RANKL-induced osteoclastogenesis was analyzed. BMMs were treated with 75 ng/mL RANKL for different periods of time ranging from 0 to 5 days. The expression of the NR1D1 was determined with RT-PCR and Western blot analysis. **d** The expression of NR1D1 during LPS-induced M1 macrophage was analyzed. BMMs were treated with 100 ng/mL LPS for different periods of time ranging from 0 to 24 h. The expression of the NR1D1 was determined with RT-PCR and Western blot analysis. **e** Tissue sections were immunohistofluorescence stained with anti-F4/80 and anti-NR1D1 antibodies (left, original magnification 20×, scale bar = 200 μm). **f** SR9009 inhibits RANKL-induced osteoclastogenesis in a concentration-dependent manner (left). BMMs were seeded in 96-well plates and cultured in complete medium containing 30 ng/mL M-CSF, 75 ng/mL RANKL, and 0, 1, 2.5, 5, or 10 μM SR9009. Trap positive cells with 3 or more nuclei per cell were counted (right). *p < 0.05, **p < 0.01 versus cells stimulated with only RANKL. **g** SR9009 abolished RANKL-induced F-actin formation. BMMs were seeded onto Osteo-Assay strips, incubated with M-CSF, RANKL, and SR9009 at the indicated concentrations for 6 days, and stained with actin-tracker green and an Alexa-labeled secondary antibody, followed by DAPI. **h** SR9009 inhibited osteoclast bone resorption (left, original magnification 4×, scale bar = 1000 μm). Osteoclasts were induced on a 0.2% collagen gel-coated six-well plate with RANKL (75 ng/mL) and M-CSF (30 ng/mL) for 6 days, digested, and seeded into Osteo-Assay strip-well plates. Osteoclasts were treated with or without SR9009 for 3 days in the presence of 30 ng/mL M-CSF and 75 ng/mL RANKL. Right panel, densitometric analysis of resorption area from three independent experiments using ImageJ software. *p < 0.05, **p < 0.01 versus cells stimulated with only RANKL. **i** SR9009 suppressed the RANKL-induced expression of NFATc1, MMP9, TRAP, c-Fos, and CTSK. BMMs were cultured with or without 10 mM SR9009 in the presence of M-CSF (30 ng/mL) and RANKL (75 ng/mL).

Proinflammatory M1 macrophages play an important role in joint inflammation and subsequent tissue damage in RA^25^. In addition, osteoclasts mediate cartilage damage and bone destruction in RA. Considering the vital role of macrophage polarization and osteoclast activity in RA, we examined whether SR9009 modulates macrophage polarization and osteoclast activity in vitro. As shown in Fig. 6a, the mRNA levels of IL-1β, IL-6, iNOS, and TNF-α were markedly decreased by SR9009 in a concentration-dependent manner in BMMs treated with LPS and IFN-γ. In addition, SR9009 increased the mRNA levels of IL-10 and Arginase-1, markers of anti-inflammatory M2 macrophages. Consistent with the decreased iNOS expression, SR9009 also inhibited M1 macrophage polarization (Fig. 6b). Western blotting indicated that with the increase of RANKL and LPS stimulation time, the protein expression of NR1D1 in BMMs decreased (Fig. 6c, d). In addition, we investigated the expression of NR1D1 in synovial macrophages of animals through a double-labeled immunofluorescence experiment. The results showed that the expression of NR1D1 in synovial macrophages of CIA mice significantly decreased, while after treatment with SR9009, the expression of NR1D1 increased (Fig. 6e). Furthermore, TRAP staining indicated that SR9009 suppressed the formation of mature osteoclasts (Fig. 6f). In addition, SR9009 also inhibited actin ring formation and osteoclastic bone resorption in vitro (Fig. 6g, h). Expression of the osteoclast markers NFATc1, MMP9, TRAP, c-Fos, and CTSK were also suppressed by SR9009 (Fig. 6i).

### NR1D1 activation ameliorates disease progression in mice with CIA

**Fig. 7:**
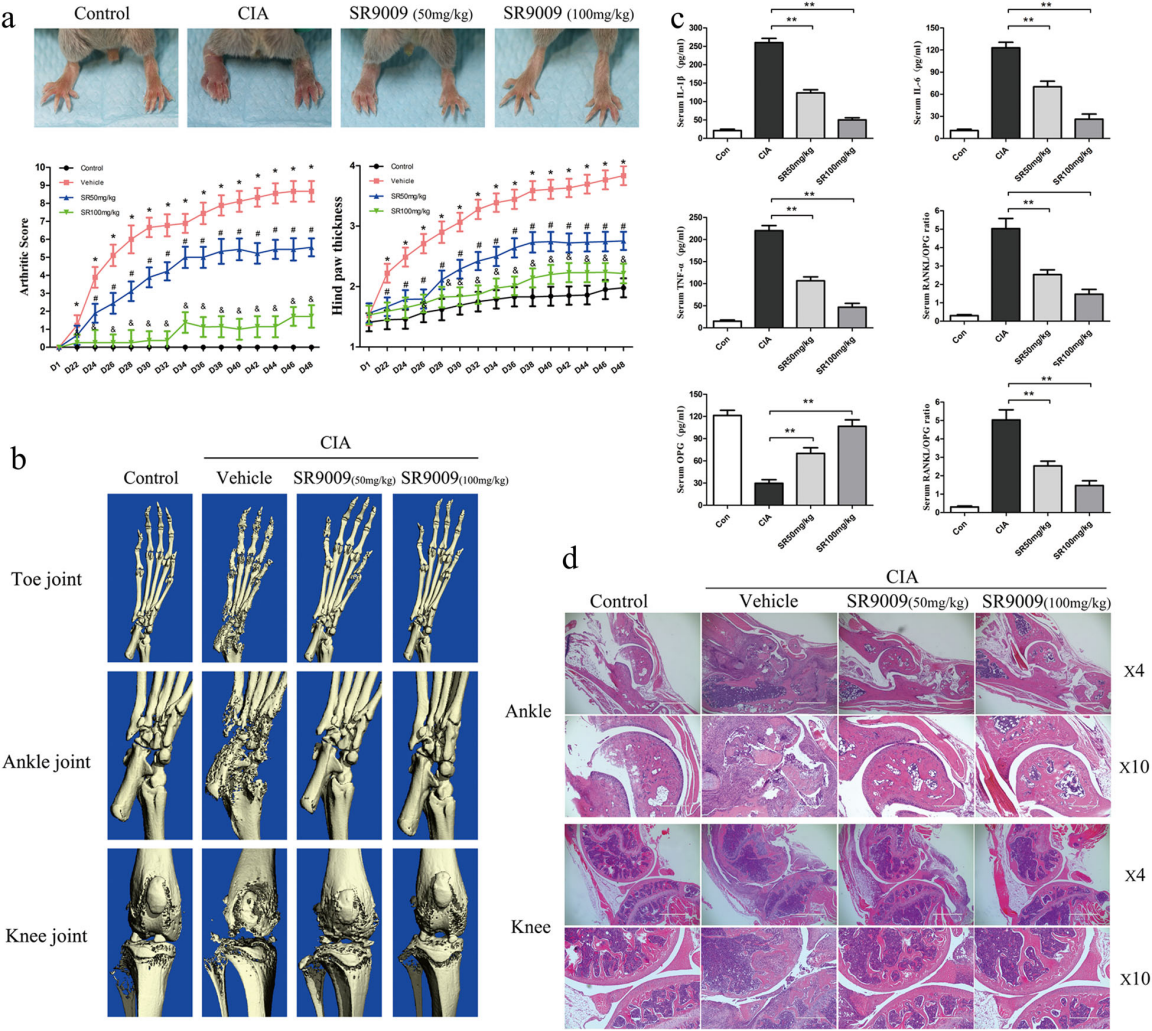
SR9009 alleviates disease severity and local bone resorption in CIA mice. **a** DBA/1 mice were immunized on days 0 and 21 and administered SR9009 every 2 days after the booster immunization. Representative hind paws on day 48. Mean arthritic score (left) and hind-paw thickness (right) were evaluated in mice with CIA administered PBS and SR9009 (50 or 100 mg/kg). Compared with the sham group, *p < 0.05; compared with the vehicle group, ^#^*p* < 0.05 or ^&^*p* < 0.01. **b** Representative micro-CT images of the hind paws of CIA mice on day 48. Data are means ± SEM, *n* = 8–10 mice per group. **c** Serum levels of proinflammatory cytokines, RANKL, and OPG in mice with CIA as determined by ELISA. Serum samples were collected on day 48 from mice with CIA and those administered SR9009. Data are means ± SEM (*n* = 8–10 mice per group). *p < 0.05, **p < 0.01 versus the vehicle group. **d** Representative images of hematoxylin-eosin staining of ankle and knee joint sections are shown (original magnification 4×, scale bar = 1000 μm or original magnification 10×, scale bar = 100 μm).

To determine whether NR1D1 activation suppresses arthritis in vivo, DBA-1 mice with CIA were established for evaluating the anti-arthritic effect of SR9009. Disease severity was evaluated every 2 days from day 21 after the initial immunization. As shown in Fig. 7a, vehicle-treated mice developed severe arthritis. However, SR9009-treated mice developed less severe arthritis, as indicated by a lower mean arthritis score and hind paw thickness, particularly in the SR9009 100 mg/kg group. µCT and 3D reconstruction were conducted to evaluate the effect of SR9009 on bone destruction. We observed that SR9009 markedly inhibited bone destruction and alleviated bone loss (Fig. 7b, d). In addition, the serum levels of IL-1β, IL-6, TNF-α, and RANKL were decreased in SR9009-treated mice compared with those in vehicle-treated CIA mice. However, the serum level of OPG, a bone-protective cytokine, was increased (Fig. 7c). This suggests that SR9009 simultaneously reduces inflammation and bone destruction in RA. Furthermore, there were no significant histopathological alterations in the liver and kidney of SR9009-treated mice compared with vehicle-treated mice (Fig. S2). These data demonstrate the safety of SR9009 treatment in mice with CIA.

### Activation of NR1D1 alleviates cartilage destruction by inhibiting the expression of MMPs and proinflammatory cytokines in vivo

**Fig. 8:**
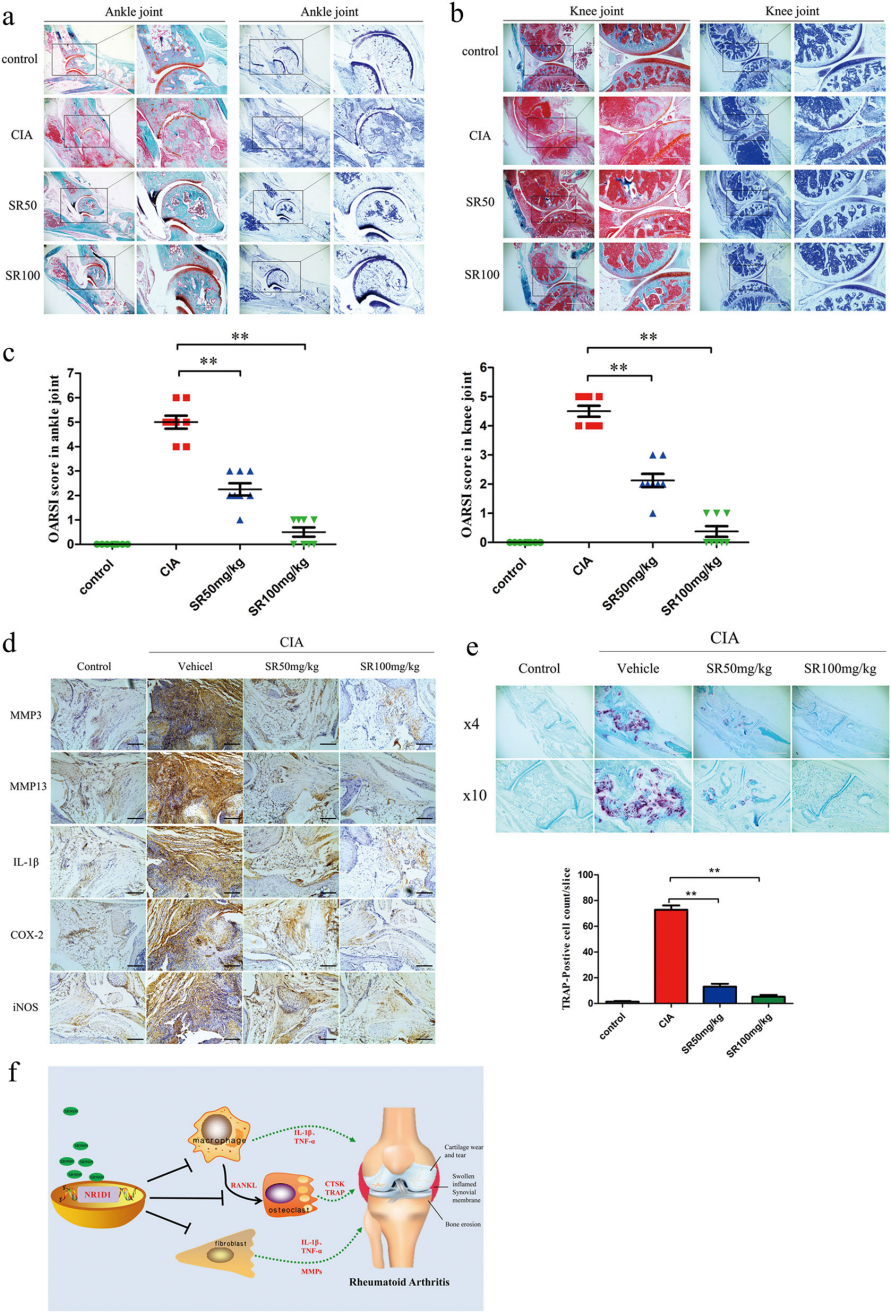
NR1D1 activation alleviates cartilage destruction by inhibiting expression of MMPs and proinflammatory cytokines in vivo. **a**, **b** Safranin-O and toluidine blue staining and **c** clinical scores of ankle-joint and knee-joint specimens. Data are means ± SEM (*n* = 8–10 mice per group). *p < 0.05, **p < 0.01 versus the vehicle group. **d** Immunohistochemical staining of MMP3, MMP13, IL-1β, iNOS, and COX2 in joint synovial tissues (original magnification 10×, scale bar = 100 μm). **e** Images of TRAP-stained ankle sections (top) and number of TRAP-positive cells (bottom). Data are means ± SEM (*n* = 8–10 mice per group). *p < 0.05, **p < 0.01 versus the vehicle group. **f** Schematic representation of NR1D1 regulates synovial inflammation and bone destruction in rheumatoid arthritis. Activation of NR1D1 reduced the expression of proinflammatory cytokines and MMPs in RA FLSs and macrophage activation and alleviated cartilage and bone destruction in rheumatoid arthritis.

Given the above, the effect of SR9009 on synovial inflammation and cartilage bone destruction was evaluated in CIA mice. Histopathological assessment of ankle and knee joint sections from SR9009-treated mice indicated that SR9009 alleviated cartilage damage, bone loss, inflammatory cell infiltration, and synovial hyperplasia. NR1D1 activation protected against ankle and knee cartilage destruction, which was supported by the significantly lower OARSI score in the SR9009 group (Fig. 8a–c). The MMPs and proinflammatory cytokines secreted by FLSs play a vital role in cartilage destruction. Therefore, we determined whether SR9009 blocked cartilage destruction by modulating MMP3, MMP13, and proinflammatory cytokine expression in the pathogenesis of CIA. As shown in Fig. 8d, expression of MMP3, MMP13, IL-1β, COX-2, and iNOS was decreased in the ankle and knee synovial tissues of SR9009-treated CIA mice but not vehicle-treated CIA mice. Furthermore, SR9009 decreased the number of TRAP-positive osteoclasts in CIA mice compared to vehicle alone (Fig. 8e).

## Discussion

RA is a chronic autoimmune disease characterized by hyperplasia of the synovial tissues and massive infiltration of diverse immune cells, which causes progressive destruction of bone and cartilage. Only a few macrophages and FLSs are present in the normal synovium, compared to the far larger numbers in the RA synovium^26^. Activation of RA FLSs stimulates continuous production of multiple proinflammatory mediators and activation of matrix proteins and degradative enzymes^27^. Activated macrophages accumulate in the synovium and release a variety of proinflammatory cytokines and proteases, which destroy joint tissues. Also, macrophages differentiate into osteoclasts, inducing massive bone resorption and driving the progression of RA^28^. Therefore, agents with anti-inflammatory and anti-bone destruction activity may have therapeutic potential for RA.

In this study, NR1D1 expression was increased in synovial tissues from patients with RA and decreased in RA FLSs stimulated by IL-1β in vitro. Also, NR1D1 activation decreased the expression of proinflammatory cytokines and MMPs, while NR1D1 silencing exerted the opposite effect. However, NR1D1 may not regulate the migration and invasion of RA FLSs (Fig. S3). Furthermore, NR1D1 activation reduced ROS generation and increased the expression of Nrf2-associated enzymes. MAPK and NF-κB pathways were blocked by the NR1D1 agonist SR9009, while those pathways were activated by NR1D1 silencing. NR1D1 activation also inhibited M1 macrophage polarization and suppressed osteoclastogenesis. SR9009 significantly suppressed cartilage damage, bone loss, inflammatory cell infiltration, and synovial hyperplasia in CIA mice. The above findings suggest a certain role for NR1D1 in the progression of RA (Fig. 8f).

Proinflammatory cytokines and MMPs play a vital role in synovial inflammation in RA, but the mechanisms responsible for the elevated levels of proinflammatory cytokines and MMPs in RA are unclear. The immune system is influenced by the circadian clock, including the core clock component NR1D1. Michael and colleagues reported that NR1D1 repressed MMP9 and CX3CR1 expression in macrophages by inhibiting enhancer-directed transcription^15^. Deletion of Rev-erbα (NR1D1) results in activation of spontaneous microglia in the hippocampus and increased levels of proinflammatory transcripts, as well as secondary astrogliosis. In addition, primary Rev-erbα^−/−^ microglia exhibit a proinflammatory phenotype and increased basal NF-κB activation^17^. NR1D1 plays a crucial role in the modulation of lung inflammation. Deletion of NR1D1 in the airway epithelium aggravated inflammation, whereas the NR1D1 ligand GSK1362 inhibited the proinflammatory genes expression in macrophages and epithelial cells and stabilized Rev-erbα protein^19^. In this study, NR1D1 expression was increased in synovial tissues from patients with RA compared to those with OA, and pharmacological activation of NR1D1 by SR9009 or overexpression of NR1D1 decreased the expression of proinflammatory cytokines and MMPs in RA FLSs. SR9009 also decreased the serum levels of proinflammatory cytokines and alleviated synovial inflammation and joint destruction in mice with CIA, consistent with the in vitro results.

ROS produced in the course of endogenous cellular oxidation play a certain role in RA^29^. Nrf2 has antioxidant and anti-inflammatory activity, and its deficiency not only accelerates the progress of arthritis and joint destruction but also promotes the production of TNF-α, IL-1β, and IL-6^30^. Sengupta and colleagues demonstrated that NR1D1 controls cellular homeostasis and reduces oxidative stress. Also, embryonic fibroblasts from C57Bl/6-SD transgenic mice showed increased mRNA levels of the downstream antioxidants HO-1, Mn superoxide dismutase, and catalase compared with wild-type mice^31^. Furthermore, Woldt and co-workers reported that NR1D1 deficiency in skeletal muscle resulted in reduced mitochondrial content and oxidative activity, whereas its overexpression in muscle or pharmacological activation in vivo increased exercise capacity^12^. Our findings support the notion that activation of NR1D1 enhances mitochondrial function against oxidative stress. ROS production was inhibited by SR9009 in RA FLSs compared with untreated cells. Moreover, pharmacological activation of NR1D1 by SR9009 or overexpression of NR1D1 increased protein levels of the Nrf2-associated enzymes HO-1 and NQO-1. Deletion of the *NR1D1* gene reduced the levels of these enzymes. SR9009 also promoted the nuclear translocation of Nrf2. Our results indicate that NR1D1 activation protect tissues from oxidative stress and inflammation by suppressing the expression of proinflammatory cytokines and MMPs in RA FLSs.

The MAPK and NF-κB pathways are implicated in the control of synovial inflammation, hyperplasia, matrix degeneration, and bone destruction. There is a close correlation between NF-κB and NR1D1^16,32^. NR1D1 regulates experimental colitis by repressing the NF-κB/NLRP3 axis^16^. In addition, Stujanna and colleagues reported that SR9009 inhibited post-MI mortality and improved cardiac function by suppressing the MAPK and NF-κB pathways^33^. Here, we found that SR9009 pretreatment suppressed IL-1β-induced phosphorylation of IKK and IκBα, as well as nuclear translocation of p65. In addition, SR9009 inhibited NF-κB transcriptional activation. Activation of NR1D1 suppressed the phosphorylation of p38 and JNK by IL-1β-stimulated RA FLSs. In turn, NR1D1 silencing activated the MAPK and NF-κB pathways.

Macrophages are key mediators of synovial inflammation because they are the main producers of proinflammatory cytokines. The role of macrophages in RA joints is usually attributed to the correlation of macrophage numbers with radiological lesions but this is reinforced by the beneficial effect of targeting macrophages and the mediators they secrete^34,35^. In addition, macrophages differentiate into osteoclasts, resulting in bone destruction^36^. As reported previously, NR1D1 modulated macrophage polarization and SR9009 inhibited osteoclastogenesis^37^. In this study, activation of NR1D1 by SR9009 decreased LPS-induced M1 polarization and promoted M2 polarization. In addition, SR9009 inhibited the formation and function of osteoclasts. These in vitro results were supported by the in vivo findings that SR9009 decreased the number of TRAP-positive cells, the serum RANKL level, and bone destruction in mice with CIA. Moreover, the histological scores and destruction of cartilage and bone were significantly decreased by SR9009, without toxicity to hepatocytes or glomerular cells.

This study has several limitations. For example, we used the NR1D1 agonist SR9009 rather than NR1D1 transgenic mice to assess the effect of NR1D1 in CIA mice. SR9009 exerts NR1D1-independent effects on proliferation, metabolism, and gene expression in two NR1D1-depleted cell types^38^. Although we demonstrated a close relationship between synovial/macrophage inflammation and NR1D1 by silencing or overexpressing NR1D1 in vitro, the effect of NR1D1 activity on NR1D1 transgenic CIA mice must be verified in vivo.

To summarize, our findings suggest that NR1D1 plays a critical role in synovial inflammation and destruction of cartilage and bone in RA. Activation of NR1D1 reduced the expression of proinflammatory cytokines in RA FLSs and macrophage activation in vitro and alleviated arthritis in vivo, suggesting NR1D1 to be a novel therapeutic target for inflammatory arthritis.

## Materials and methods

### Reagents and antibodies

SR9009 was obtained from Shanghai Lollane Biological Technology (Shanghai, China). Bovine type II collagen and complete Freund adjuvant were purchased from Chondrex (Redmond, WA, USA). Recombinant murine soluble receptor activated of NF-κB ligand (RANKL) and macrophage colony-stimulating factor (M-CSF) were obtained from R&D Systems (Minneapolis, MN, USA). Cell Counting Kit-8 (CCK8) kits were obtained from Boster Biotechnology (Wuhan, China). IL-1β was obtained from PeproTech (Rocky Hill, NJ, USA). Lipofectamine 3000 reagent was purchased from Invitrogen (Carlsbad, CA, USA). Anti-p38, p-p38, extracellular signal-regulated kinase (ERK), p-ERK, c-Jun N-terminal kinase (JNK), p-JNK, IκBα, p-IκBα, p65, p-p65, IκB kinase (IKK)-β, IKKα, p-IKKα/β, NR1D1, and c-Fos antibodies were purchased from Cell Signaling Technology (Beverly, MA, USA). Anti-tartrate-resistant acid phosphatase (TRAP), MMP9, MMP3, MMP13, cathepsin K (CTSK), iNOS, F4/80, and nuclear factor of activated T cells 1 (NFATc1) antibodies were obtained from Abcam (Cambridge, UK). Anti-heme oxygenase-1 (HO-1), NAD(P)H dehydrogenase [quinone] 1 (NQO-1), Kelch-like ECH-associated protein 1 (KEAP1), Nrf2, IL-1β, and cyclooxygenase (COX)-2 antibodies were purchased from Proteintech Group (Wuhan, China). The TRAP staining kit and LPS were obtained from Sigma-Aldrich (St. Louis, MO, USA). The Osteo-Assay surface was acquired from Corning Incorporated Life Science (Corning, NY, USA). Reactive oxygen species (ROS) kit and Annexin V-fluorescein isothiocyanate/propidium iodide (PI) apoptosis kits were obtained from Beyotime Institute of Biotechnology (Jiangsu, China). The basal culture medium was obtained from Invitrogen.

### Isolation and culture of primary FLSs

Human RA FLSs were obtained from patients with RA who met the 2010 RA criteria and had undergone total knee arthroplasty or synovectomy^39,40^. Primary synovial tissues from patients with osteoarthritis (OA) were used as the negative control. In this study, twenty-eight pairs of fresh synovial tissues were collected from patient who underwent surgical resection in tongji hospital. Briefly, the synovial tissue was cut into 1–2 mm^3^ pieces, digested with dipase and collagenase, and plated evenly in a culture dish. After 6 h, liberated cells were cultured in Dulbecco’s modified Eagle’s medium (DMEM) containing 10% fetal bovine serum (FBS) in a standard humified incubator and FLSs between the fourth and eighth generations were used. All of the procedures involving human specimens were performed under protocols approved by the Ethics Committee of Tongji Hospital.

### Cell viability assay

RA FLSs were plated in 96-well plates and incubated with vehicle or SR9009 (1–10 μM) for 12, 24, 48, or 72 h. All tests were performed in triplicate and cell viability was assayed using a CCK-8 kit according to the provided instructions. After the cells were incubated for 1 h, the absorbance at 450 nm was measured using a microplate reader.

### Flow cytometry

To analyze apoptosis, RA FLSs were exposed to vehicle or SR9009 (1–10 μM) for 24 h. Approximately 5 × 10^5^ cells were collected and stained with an Annexin V-fluorescein isothiocyanate/propidium iodide (PI) apoptosis kit according to the manufacturer’s instructions for analysis by flow cytometry (BD Accuri™ C6; BD Biosciences, San Jose, CA, USA). Data were processed by FlowJo software (Treestar, Inc., San Carlos, CA, USA).

### Small interfering RNA and plasmid transfections

Transfection of NR1D1 small interfering RNA (siRNA) or control siRNA (RiboBio, Guangzhou, China) was conducted using Lipofectamine 3000 reagent (Invitrogen). In six-well plates, 50 pM siRNA, 5 μL of Lipofectamine 3000, 250 µL of Opti-MEM, and 2 mL of complete medium were mixed. The controls consisted of scrambled siRNAs. Cells were transfected with an NR1D1-encoding plasmid (GeneCopoeia, EX-A0803-M02-B, Rockville, MD, USA) using Lipofectamine 3000 (Invitrogen), according to the manufacturer’s instructions. The siRNA sequences are listed in Supplemental Table 1. Knockdown and overexpression efficiency was determined by qPCR and western blotting.

### Animals

Forty male DBA/1 mice (8–10 weeks of age) were purchased from the Model Animal Research Center of Nanjing University (Nanjing, China). All animals were maintained under standard conditions (room temperature 25 °C, relative humidity 45–50%, and a 12/12-h dark/light cycle) in the Animal Research Center of Huazhong University of Science and Technology. Water and food were provided ad libitum. All animal experimental procedures and care were approved by the Ethics Committee and the Institutional Animal Research Committee of Tongji Medical College.

### Collagen-induced arthritis and SR9009 treatment

Bovine type II collagen (2 mg/mL in 0.05 M acetic acid; Chondrex, Inc., Redmond, WA, USA) was mixed with the same volume of Freund complete adjuvant (2 mg/mL *Mycobacterium tuberculosis*; Chondrex, Inc.). DBA/1 mice were given an intradermal injection of 100 μg of bovine type II collagen on day 1 into the base of the tail. On day 21, mice received a booster intradermal injection of 100 μg of bovine type II collagen emulsified in an equal volume of incomplete Freund adjuvant (Chondrex, Inc.). The mice were randomly divided into control, collagen-induced arthritis (CIA), SR9009 low-dose, and SR9009 high-dose groups. To evaluate the effect of SR9009 on the development of arthritis, mice were administered intraperitoneally 50 or 100 mg/kg body weight SR9009 (low and high doses, respectively) seven times per week for 4 weeks after the booster injection. At 48 days, the animals were anesthetized and euthanized.

### Clinical assessment of arthritis

The incidence, severity and progression of arthritis were assessed every 2 days after booster immunization by determining the arthritis score and measuring posterior paw thickness and body weight. The paws were assigned clinical scores as follows: 0—normal, 1—mild swelling of the ankle or wrist without deformities and erythema, 2—moderate redness and swelling of the ankle or wrist, 3—severe redness and swelling of the entire paw including the digits, and 4—severe erythema and swelling of the limb or ankylosis^41^. Swelling of the paw was evaluated by measuring paw thickness using vernier calipers. The clinical evaluation of arthritis was determined by two rheumatologists in an unknown experimental group based on the above scoring criteria.

### Micro-computed tomography

After euthanasia, the hind paws of the mice were fixed in 4% formaldehyde, and the knee and ankle joints and paws were analyzed by high-resolution micro-computed tomography (μCT). The images were reconstructed into three-dimensional (3D) by using the manufacturer’s software and the voxel size is 9 μm.

### Histopathological assessment

The ankle and knee joints of the mice were fixed in paraformaldehyde and decalcified for 4 weeks. For histological assessment, samples were embedded in paraffin and sectioned at 5 μm thickness. Tissue sections stained with hematoxylin and eosin (HE) and Safranin O-fast green were scored for synovial inflammation and cartilage and bone destruction as follows: 0 (no significant change), 1 (mild change), 2 (moderate change), and 3 (severe change)^42^. In addition, the degree of articular cartilage damage was assessed using the modified International Osteoarthritis Research Association (OARSI) scoring system^43^. Sections were stained with commercial kits and count trap-positive cells under the microscope (Sigma-Aldrich).

### Immunocytochemical analysis

Tissue samples were sectioned at 5 mm thickness. The sections were prepared for antigen restoration by immersion in 5% hydrogen peroxidase at 37 °C for 10 min, and incubated overnight at 4 °C with primary antibodies against MMP13 (1:100), MMP3 (1:100), IL-1β (1:100), iNOS (1:100), COX-2 (1:200), or NR1D1 (1:200). The sections were incubated with a suitable secondary antibody and counterstained with hematoxylin.

### Enzyme-linked immunosorbent assay (ELISA)

Sera was collected on day 48 and stored at −80 °C until use. Serum TNF-α, IL-6, IL-8, IL-1β, RANKL, and osteoprotegerin (OPG) levels were measured by ELISA according to the manufacturer’s instructions (Boster Biotechnology).

### Electrophoretic mobility shift assay (EMSA)

RA FLSs were pretreated with DMSO or 10 μM SR9009 for 24 h and stimulated or not with IL-1β (10 ng/mL) for 30 min. Nuclear extracts were prepared using nuclear protein extraction kits (Beyotime Institute of Biotechnology). Equal amounts of nuclear extracts were incubated with an NF-κB probe in reaction buffer at room temperature for 30 min. The mixture was separated in a 6.5% polyacrylamide gel and transferred onto a positively charged nylon membrane. DNA was cross-linked by an ultraviolet cross-linker and the membrane was blocked and incubated with streptavidin–horseradish peroxidase conjugate.

### Measurement of the ROS level

The cellular ROS level was measured using 2′,7′-dichlorofluorescin diacetate (DCFH-DA; Sigma Aldrich). In brief, RA FLSs were incubated with SR9009 in 12-well plates and stimulated with IL-1β (10 ng/mL) for 24 h. Next, RA FLSs were incubated with DCFH-DA (10 μM) for 30 min at room temperature in the dark and washed four times with serum-free medium and one time with phosphate-buffered saline (PBS). ROS levels were analyzed by fluorescence microscopy.

### Culture of bone marrow macrophages and macrophage polarization

Bone marrow macrophages (BMMs) were isolated from mice as described above^44^. Briefly, BMMs were isolated from long bones and cultured for 24 h in α-MEM/10% FBS and 30 ng/mL M-CSF. The cells were continuing cultured for 3 days in the same medium to generate BMMs after removing adherent cells. M1 macrophages were induced from BMMs with 100 ng/mL LPS (Sigma-Aldrich) and 10 ng/mL interferon (IFN)-γ (PeproTech), and M2 macrophages with 20 ng/mL IL-4 (PeproTech). The cells were treated with LPS and IFN-γ with or without SR9009 (5 or 10 μM) and subjected to immunofluorescence staining as described above.

### In vitro osteoclastogenesis assay

BMMs were plated in 96-well plates at a density of 2 × 10^4^/well overnight. The following day, BMMs were stimulated with M-CSF (30 ng/mL) and RANKL (75 ng/mL) in the presence or absence of SR9009 (0, 1, 2.5, 5, or 10 μM). Medium was replaced daily until osteoclasts formed. The cells were fixed in 4% paraformaldehyde for 30 min and stained for TRAP activity using a TRAP staining kit. TRAP positive cells with three or more nuclei were identified as mature osteoclasts.

### Staining of podosome belts and pit-formation assay

To visualize podosome belts, BMMs were plated onto the Osteo-Assay strips and stimulated with SR9009 (0, 1, 2.5, 5, or 10 μM). Osteoclasts were next fixed, permeabilized, blocked and stained with actin-tracker for 1 h at room temperature and counterstained with DAPI for 10 min. Images were captured using a fluorescence microscope and actin ring formation was analyzed with ImageJ software (National Institutes of Health, Bethesda, MD, USA).

To determine whether SR9009 affects osteoclast function, BMMs were inoculated onto a 0.2% collagen gel-coated 6-well plate with RANKL (75 ng/mL) at a density of 5 × 10^5^/well for 4 days. Next, the cells were digested and seeded onto an Osteo-Assay surface in a multiwell plate and incubated with SR9009 for 48 h in complete α-MEM containing 75 ng/mL RANKL and 30 ng/mL M-CSF. Next, the wells were washed with 5% sodium hypochlorite disinfectant to remove cells and images were captured and reabsorption area quantified using ImageJ software. The reabsorption area per well and the percentage reabsorption area per osteoclast were calculated to quantify the activity of osteoclasts.

### Protein extraction and western blotting

Total protein was extracted using radioimmunoprecipitation assay (RIPA) lysis buffer (Beyotime, Shanghai, China) supplemented with protease inhibitors and phosphatase inhibitor cocktail (Boster Biotechnology). The nuclear and cytoplasmic protein was extracted using a NE-PER^TM^ nuclear and cytoplasmic extraction reagent kit (Beyotime, Shanghai, China). Equal amounts of protein were separated by 10% sodium dodecyl sulfate-polyacrylamide gel electrophoresis (SDS-PAGE) and transferred to a polyvinylidene fluoride (PVDF) membrane. The PVDF membrane was blocked with 5% skim milk in a Tris-buffered saline with 0.1% Tween 20 buffer (TBST) and incubated with the corresponding primary antibodies overnight at 4 °C. After three washes with TBST, the membrane was incubated with the appropriate horseradish peroxidase-conjugated secondary antibodies for 60 min. Finally, an enhanced chemiluminescence detection system (Thermo Fisher Scientific, Waltham, MA, USA) and ChemiDoc Touch Imaging System (Bio-Rad Laboratories, Hercules, CA, USA) were used to identify antibody-bound proteins. Protein levels were quantified using ImageJ software and normalized to the control.

### RNA extraction and qRT-PCR

Total RNA was isolated from cultured RA FLSs or BMMs using TRIzol reagent (Thermo Fisher Scientific) according to the manufacturer’s specifications. mRNA (1 μg) was used to synthesize cDNA according to the manufacturer’s instructions (Toyobo, Osaka, Japan). mRNA levels were determined by qRT-PCR as described in the SYBR Green Quantitative PCR Protocol (TaKaRa, Shiga, Japan). The primer sequences are listed in Supplemental Table 2.

### Cell scratch and migration and invasion assay

RA FLSs were cultured in a 12-well plate to complete confluence. Then, gently scratch the cells with a 200 µL micropipette tip in the center of the well and incubate with serum-free medium. Images were obtained at 0 and 24 h after scratching. Quantify the width of wound healing and compare it to baseline values.

For migration and invasion assay, RA FLSs were suspended in 200 μL serum-free medium at a concentration of 6 × 10^4^ cells/mL and plated in the upper wells of Boyden chambers (8.0 μm pore, Corning, USA) with (invasion) or without (migration) Matrigel (BD Bioscience, USA). The bottom wells were added 600 μL DMEM medium with 10% FBS. After 24 h of incubation, the upper chamber was fixed with methanol and then stained with 0.1% crystal violet. Photograph and count cells that migrate or invade the back of the chamber. Select five random fields to calculate migrating or invading cells.

### Immunohistofluorescence

Tissue samples were sectioned at 5 mm thickness. The sections were incubated overnight at 4 °C with primary antibodies against F4/80 (1:100) and NR1D1 (1:100). The sections were incubated with a suitable Goat Anti-Rat IgG H&L (Alexa Fluor 488) or Goat Anti-Rabbit IgG H&L (Alexa Fluor 647) for 30 min and counterstained with DAPI. Images were captured using a fluorescence microscope.

### Statistical analysis

The data are presented as means ± SEM. Statistical analysis was performed using Prism software (version 6.0; GraphPad Software). All experimental data were independent repeated at least three times. Statistical evaluation involved one-way analysis of variance followed by the Tukey test for multiple comparisons. *p* < 0.05 was considered statistical significance.

## Supplementary information

Supplemental Table 1. siRNA Sequences.

Supplemental Table 2. Primers for qPCR.

Supplemental Fig 1.SR9009 did not significantly inhibit the proliferation of RA FLS cells.

Supplemental Fig 2. Effect of SR9009 on the liver and kidney of CIA mice.

Supplemental Fig 3. NR1D1 may not regulate the migration and invasion of RA FLSs.

Supplemental figure legneds
