# Supplementary material for: NR1D1 modulates synovial inflammation and bone destruction in rheumatoid arthritis
Source: Cell Death Dis. 2020 Feb 18;11(2):129. doi: 10.1038/s41419-020-2314-6 (PMC7028921; doi:10.1038/s41419-020-2314-6)
Supplement: Supplementary file 1 — Supplemental Table 1. siRNA Sequences. [file 41419_2020_2314_MOESM1_ESM.docx]

**Supplemental Table 1. SiRNA Sequences**

| Target | Sense | Antisense |
| --- | --- | --- |
| NR1D1 001 | CAUGUCCUAUGAACAUGUAdTdT | UACAUGUUCAUAGGACAUGdTdT |
| NR1D1 002 | GCAACUCAAAGAAUGUUCUdTdT | AGAACAUUCUUUGAGUUGCdTdT |
| NR1D1 003 | GUGCGCUUUGCUUCGUUGUdTdT | ACAACGAAGCAAAGCGCACdTdT |
