# Supplementary material for: NR1D1 modulates synovial inflammation and bone destruction in rheumatoid arthritis
Source: Cell Death Dis. 2020 Feb 18;11(2):129. doi: 10.1038/s41419-020-2314-6 (PMC7028921; doi:10.1038/s41419-020-2314-6)
Supplement: Supplementary file 2 — Supplemental Table 2. Primers for qPCR. [file 41419_2020_2314_MOESM2_ESM.docx]

**Supplemental Table 2. Primers for qPCR**

| Target gene | Species | Forward | Reverse |
| --- | --- | --- | --- |
| GAPDH | Human | TGTGGGCATCAATGGATTTGG | ACACCATGTATTCCGGGTCAAT |
| NR1D1 | Human | TGGACTCCAACAACAACACAG | GATGGTGGGAAGTAGGTGGG |
| IL-1β | Human | ATGATGGCTTATTACAGTGGCAA | GTCGGAGATTCGTAGCTGGA |
| IL-6 | Human | ACTCACCTCTTCAGAACGAATTG | CCATCTTTGGAAGGTTCAGGTTG |
| IL-8 | Human | ACTGAGAGTGATTGAGAGTGGAC | AACCCTCTGCACCCAGTTTTC |
| iNOS | Human | TTCAGTATCACAACCTCAGCAAG | TGGACCTGCAAGTTAAAATCCC |
| COX-2 | Human | TAAGTGCGATTGTACCCGGAC | TTTGTAGCCATAGTCAGCATTGT |
| MMP-3 | Human | AGTCTTCCAATCCTACTGTTGCT | TCCCCGTCACCTCCAATCC |
| MMP-13 | Human | ACTGAGAGGCTCCGAGAAATG | GAACCCCGCATCTTGGCTT |
| CXCL10 | Human | GTGGCATTCAAGGAGTACCTC | TGATGGCCTTCGATTCTGGATT |
| CCL2 | Human | CAGCCAGATGCAATCAATGCC | TGGAATCCTGAACCCACTTCT |
| GAPDH | Mouse | ACGGGAAGCTCACTGGCATGGCCTT | CATGAGGTCCACCACCCTGTTGCTG |
| IL-1β | Mouse | GAAATGCCACCTTTTGACAGTG | TGGATGCTCTCATCAGGACAG |
| IL-6 | Mouse | TTCACAAGTCGGAGGCTT | CAGTTTGGTAGCATCCAT |
| IL-10 | Mouse | GCCTTGCAGAAAAGAGAGCT | AAAGAAAGTCTTCACCTGGC |
| TNF-α | Mouse | CAGGCGGTGCCTATGTCTC | CGATCACCCCGAAGTTCAGTAG |
| arginase-1 | Mouse | CCAGAAGAATGGAAGAGTGT | GCAGATATGCAGGGAGTCACC |
