# Supplementary figures and images for: NR1D1 modulates synovial inflammation and bone destruction in rheumatoid arthritis
Source: Cell Death Dis. 2020 Feb 18;11(2):129. doi: 10.1038/s41419-020-2314-6 (PMC7028921; doi:10.1038/s41419-020-2314-6)

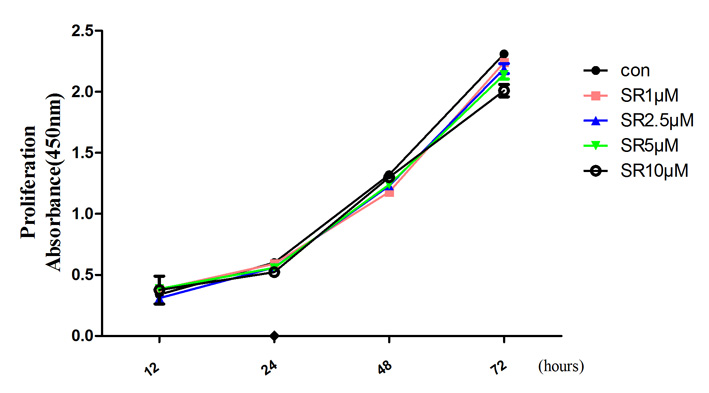

Supplement: Supplementary file 3 — Supplemental Fig 1.SR9009 did not significantly inhibit the proliferation of RA FLS cells. [file 41419_2020_2314_MOESM3_ESM.png]

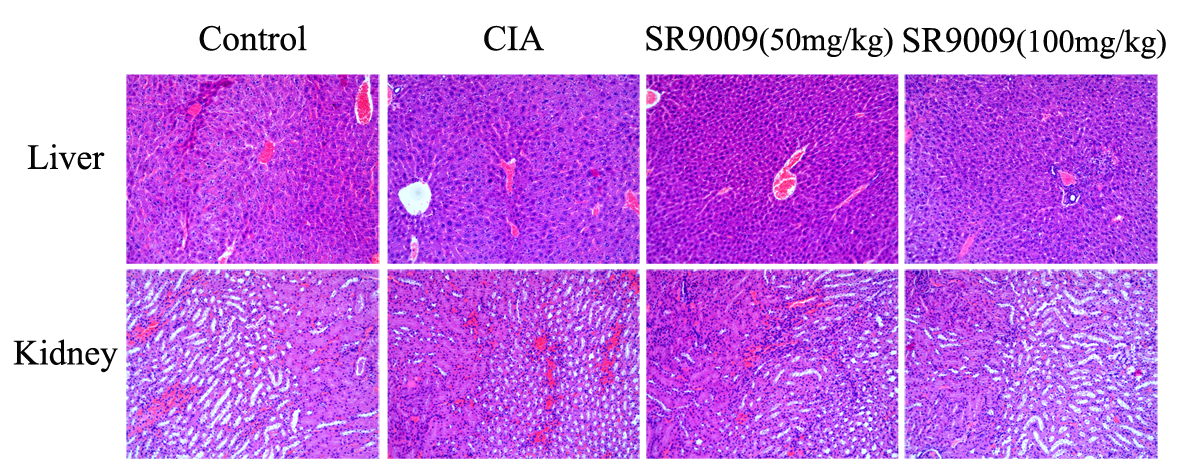

Supplement: Supplementary file 4 — Supplemental Fig 2. Effect of SR9009 on the liver and kidney of CIA mice. [file 41419_2020_2314_MOESM4_ESM.png]

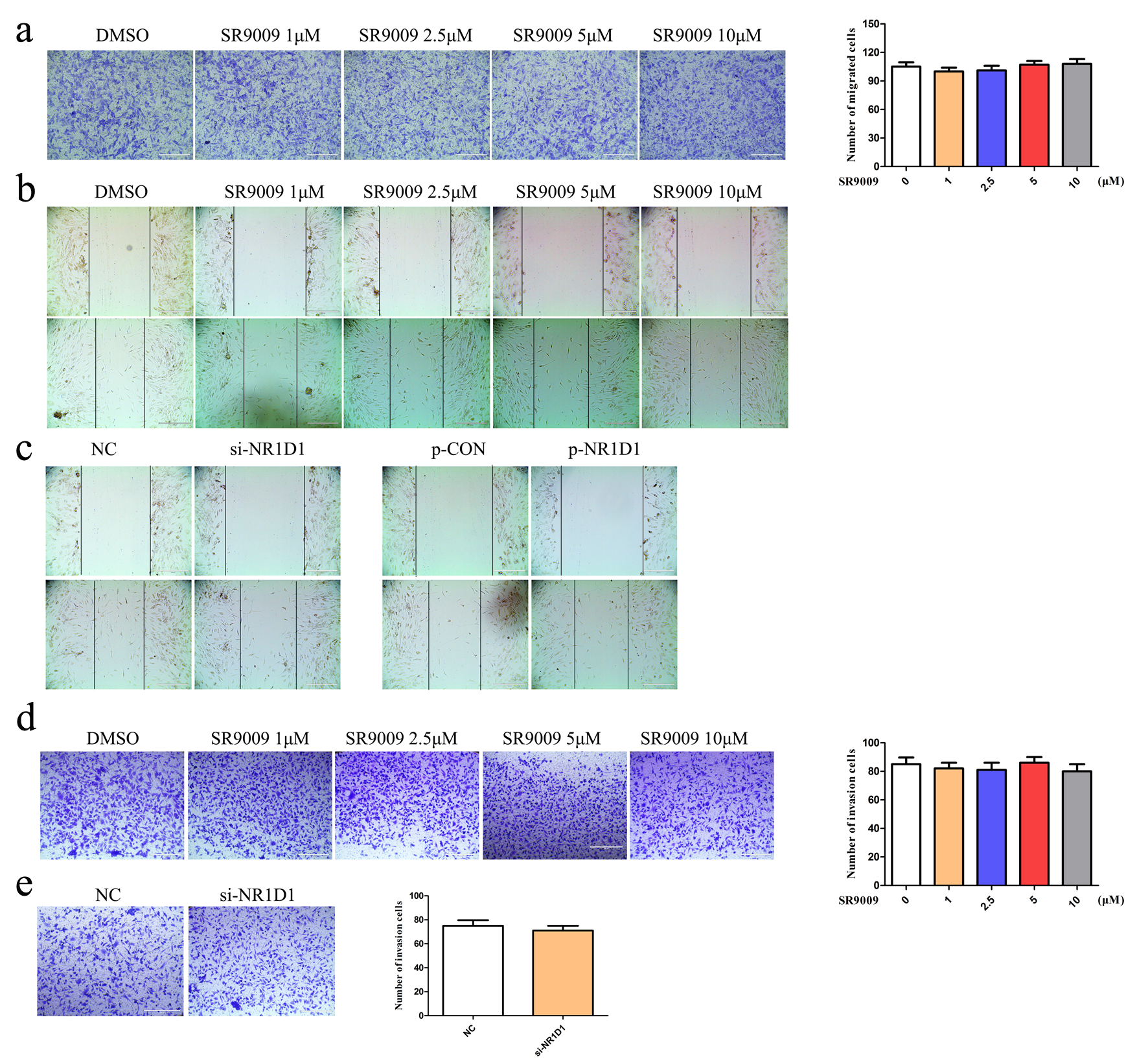

Supplement: Supplementary file 5 — Supplemental Fig 3. NR1D1 may not regulate the migration and invasion of RA FLSs. [file 41419_2020_2314_MOESM5_ESM.png]
