# Supplementary material for: NR1D1 modulates synovial inflammation and bone destruction in rheumatoid arthritis
Source: Cell Death Dis. 2020 Feb 18;11(2):129. doi: 10.1038/s41419-020-2314-6 (PMC7028921; doi:10.1038/s41419-020-2314-6)
Supplement: Supplementary file 6 — Supplemental figure legneds [file 41419_2020_2314_MOESM6_ESM.docx]

**Supplemental Fig 1.** RA FLS cells were treated with various concentrations of SR9009 (0, 1, 2.5, 5 and 10 μM) for different periods of time (12, 23, 48, and 72 hours). RA FLS cells proliferation was assessed using a cell counting kit at 12, 23, 48, and 72 hours days after treatment. SR9009 did not significantly inhibit the proliferation of RA FLS cells.

**Supplemental Fig 2.** **Effect of SR9009 on the liver and kidney of CIA mice.** Photomicrographs show the histopathology of the liver and kidney fromVehicle- or SR9009-treated mice. Original magnification 20×.

**Supplemental Fig 3.** NR1D1 may not regulate the migration and invasion of RA FLSs.(a) Effect of NR1D1 agonist SR9009 on the migration of RA FLSs. Quantitative analyses (right) and representative images (left, original magnification 10×, scale bar = 400 μm). Data are means ± SEM of three independent experiments. *p < 0.05, **p < 0.01 *versus* control. (b) Effect of NR1D1 agonist SR9009 on the migration of RA FLSs after wounding (original magnification 10×, scale bar = 400 μm). (c) Effect of NR1D1 inhibition or activation on the migration of RA FLSs after wounding (original magnification 10×, scale bar = 400 μm). (d) Effect of NR1D1 agonist SR9009 on the invasion of RA FLSs. (e) Effect of NR1D1 inhibition on the invasion of RA FLSs. Quantitative analyses (right) and representative images (left, original magnification 10×, scale bar = 400 μm). Data are means ± SEM of three independent experiments. *p < 0.05, **p < 0.01 *versus* control.
